# Fire-Needle Moxibustion for the Treatment of Knee Osteoarthritis: A Meta-Analysis

**DOI:** 10.1155/2016/1392627

**Published:** 2016-06-15

**Authors:** Yidan Wang, Xiaohua Xie, Xiaoyue Zhu, Minjie Chu, Yihua Lu, Tian Tian, Xun Zhuang, Liying Jiang

**Affiliations:** ^1^Department of Epidemiology, School of Public Health, Nantong University, Nantong, Jiangsu 226009, China; ^2^Department of Endodontics, Institute of Hard Tissue Development and Regeneration, The Second Affiliated Hospital of Harbin Medical University, Harbin, Heilongjiang 150001, China

## Abstract

*Objectives*. The aim of this study was to evaluate the effectiveness of fire-needle moxibustion as an intervention in the treatment of knee osteoarthritis (KOA).* Methods*. An updated meta-analysis of randomized controlled trials (RCTs) on fire-needle moxibustion in treating KOA was conducted by searching PubMed, Embase, the Cochrane Library, Web of Science, Wanfang database, and the Chinese Medical Database (CNKI) since their inception through March 2016. The meta-analysis was performed using RevMan 5.3.* Results*. Thirteen RCTs were identified in the systematic study which consisted of 1179 participants. Fire-needle moxibustion treatment group had a statistical significance on recovery rate as well as recovery and marked-improvement rate compared with control group. Subgroup analysis indicated that there was significant difference between fire-needle moxibustion group and control group. However, GRADE analysis indicated that the quality of evidence for all outcomes was relatively low. Only two of 13 studies reported adverse reactions (difficulty in movement and intolerance of cold).* Conclusion*. This meta-analysis suggests that fire-needle moxibustion is more effective than control group in symptom management of KOA. Further high quality trials should be conducted to evaluate the effectiveness of fire-needle moxibustion on KOA.

## 1. Introduction

Osteoarthritis (OA), the most common joint bone disease, is characterized by the chronic degenerative changes of joint structure including cartilage surfaces and subchondral bone [1]. As the major part of weight-bearing peripheral joint, the knee is the most frequently affected site [2]. The structural changes result in pain, stiffness, swelling, and tenderness and then reduce physical function and affect the quality of life of patients [3]. About 10–13% of the causes of disability for people aged 60 years and above are attributable to knee osteoarthritis (KOA) [4]. It has become a major public health problem as KOA is associated with advancing age and universal obesity [5, 6].

The occurrence and development of OA are related to multiple aetiological factors such as genetics, environmental factors, and biomechanical components. Presently, the symptomatic approach is clinically the premier choice [7, 8]. The conventional treatment of KOA includes medicine and surgical treatment [9]. Although these conventional treatments are usually recommended for the relief of pain, severe adverse events attached to these therapies were reported [10, 11]. Acupuncture and moxibustion, as mainstream complementary and alternative treatments in nonpharmacological therapy, have been widely accepted by patients across the world to relieve pain, improve function, and restrain the progression of KOA [12].

**Figure 1:**
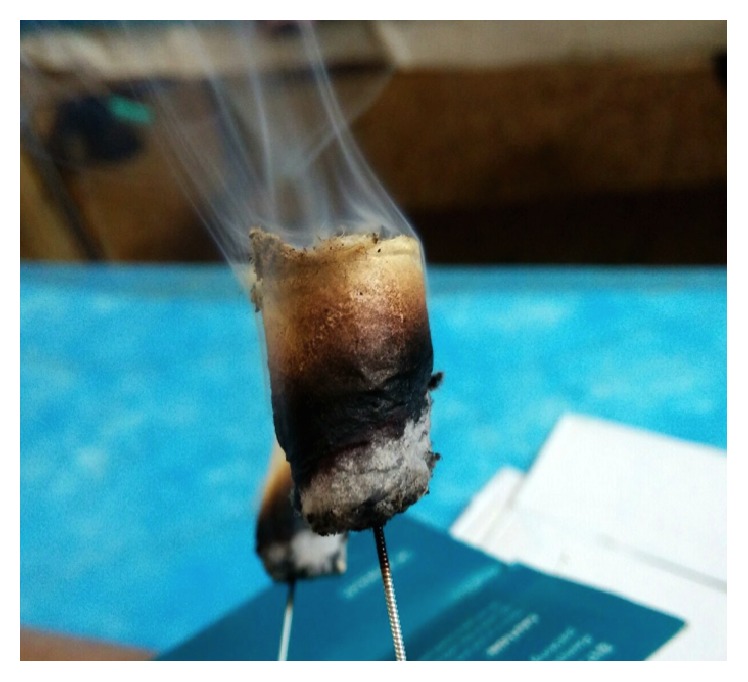
The photo of warm-needle moxibustion.

**Figure 2:**
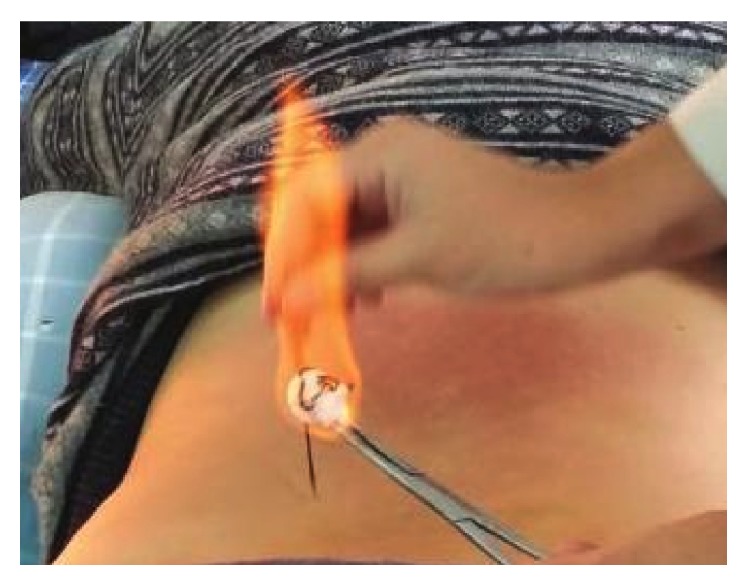
The photo of fire-needle moxibustion.

Filiform-needle acupuncture, warm-needle moxibustion, and fire-needle moxibustion are three main types of acupuncture and moxibustion. Topical acupoints such as Heding, Xiyan, Xuehai, Liangqiu, and Ashi were usually selected. Filiform-needle acupuncture is a modality of traditional acupuncture applied directly to the skin surface with sterile stainless steel [13]. Warm-needle moxibustion involves stimulating acupuncture points with burning moxa containing the herb* Artemisia vulgaris* [14] (Figure 1). Fire-needle moxibustion is an ancient method of external therapy that combines acupuncture with moxibustion. The acupoints of fire-needle therapy will be the same as those in the filiform needle and warm needle except for needling depth, acupuncture manipulation, and needle retention time. The tip of the fire needle is round so as to increase the contact area with the lesions. What is more, the fire needle has the property of high temperature resistance (Figure 2). In the operation, needle retention time of the fire needle is less than that of filiform needle and warm needle. Skilled Traditional Chinese Medicine (TCM) practitioners use the needle to prick the selected acupoints by depth of 0.3–0.5 cun and then remove the needle swiftly. Unless the needle holes are bleeding, practitioners are not supposed to press [15].

At present, filiform-needle acupuncture and warm-needle moxibustion have been confirmed as effective treatments of KOA [16, 17]. Several randomized controlled trials (RCTs) have proved that fire-needle moxibustion could relieve pain and improve the prognosis of KOA. However, the efficacy of fire-needle moxibustion has not been systematically assessed [18]. Therefore, we undertook a systematic review and meta-analysis to assess the current evidence for the effect and safety of fire-needle moxibustion in treating OA.

## 2. Methods

### 2.1. Search Strategy

An Internet-based search was performed through PubMed, Embase, the Cochrane Library, Web of Science, Wanfang database, and the Chinese Medical Database (CNKI) since their inception through March 2016. The publication language was limited to English and Chinese. Search terms in Chinese included “Huo Zhen”, “gu guan jie yan”, and “xi gu guan jie yan”. The following search terms in English were used: “fire needling”, “fire needle”, “osteoarthritis”, “OA”, “arthritis”, “joint disease”, and the corresponding free terms. The search was restricted to studies of human participants.

### 2.2. Inclusion and Exclusion Criteria

Inclusion criteria were as follows. (1) Types of study: studies were eligible if they were RCTs. (2) Types of participants: the research samples included participants in accordance with the KOA diagnosis criteria from the American College of Rheumatology (ACR) and the Guiding Principles of Clinical Research on New Drugs for TCM [19, 20]. (3) Types of interventions: studies that compared fire-needle moxibustion with routine acupuncture or warm-needle moxibustion were eligible. No restriction was made regarding selection of acupoints. (4) Outcome measure: clinical recovery rate and recovery and marked-improvement rate were the major outcome of assessment. Their pain intensity was assessed according to the Visual Analog Scale (VAS), Index of Severity for Osteoarthritis (ISOA), and/or the scale of the Western Ontario and McMaster Universities Arthritis Index (WOMAC). If available, safety served as the secondary outcome.

The following exclusion criteria were applied: (1) the study was of nonrandomized or uncontrolled trials; (2) the study did not describe intervention methods clearly; (3) the study was lacking sample size and data of related index; (4) the study or data of the research was reported repeatedly.

### 2.3. Data Extraction

Two reviewers (Yidan Wang and Xiaoyue Zhu) evaluated the obtained studies independently according to a preconfigured form. Characteristics of the included studies such as date of publication, author, participants' quantity, interventions, outcome measures, and results were presented in Table 1. Disagreements were resolved by discussion with a third reviewer (Liying Jiang).

### 2.4. Risk of Bias and Quality Assessment

The Cochrane Risk of Bias Tool was used to evaluate the methodological quality of the included studies (version 5.1.0) [21]. The tool included seven domains: random sequence generation, allocation concealment, blinding of participants and personnel, blinding of outcome assessment, incomplete outcome data, selective report, and other sources of bias. Each item was judged by the following criteria: low (low risk of bias), unclear (uncertain risk of bias), and high (high risk of bias). Two reviewers (Yidan Wang and Xiaoyue Zhu) checked the aspects of each included study independently. GRADEpro 3.6.1 software was applied to assess the quality of evidence for all outcomes, and the results were summarized in Table 2.

### 2.5. Statistical Analysis

All statistical analyses were performed by using Review Manager version 5.3 (the Nordic Cochrane Centre, the Cochrane Collaboration, Copenhagen, Denmark). For dichotomous outcomes, we calculated relative risk (RR) with 95% confidence interval (CI). For continuous outcomes, standardized mean difference (SMD) with 95% confidence interval (CI) was used to present the effect size. Heterogeneity was assessed by applying a chi-squared test. *I* ^2^ was considered to indicate a substantial level of heterogeneity. A random-effects model was used if *I* ^2^ > 50%. We constructed the funnel plot by Stata 12.0 to assess the potential publication bias when 10 or more trials were included in the meta-analysis [22]. A subgroup analysis should be conducted because different types of pain scale would lead to statistical heterogeneity [23].

## 3. Results

### 3.1. Study Selection and Characteristics

**Figure 3:**
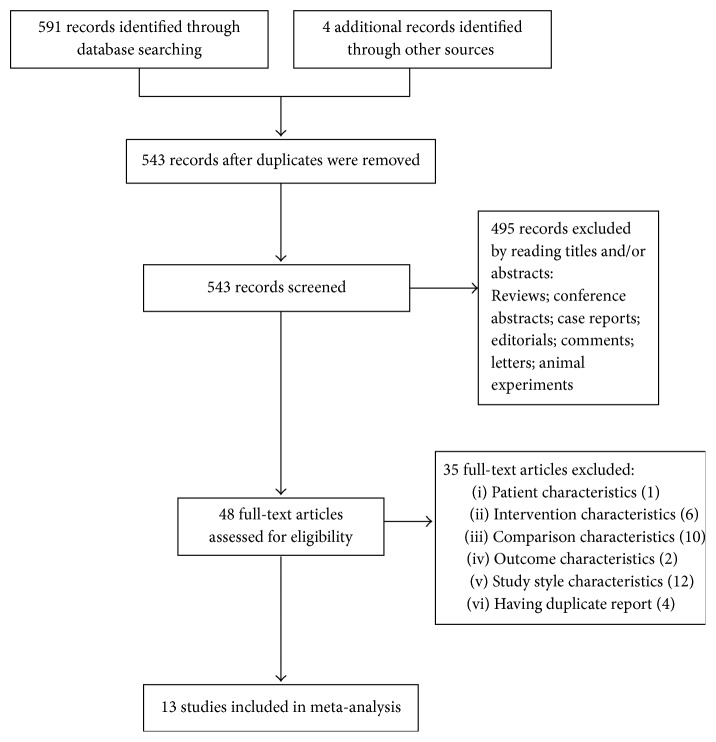
Flowchart of study search.

A flowchart of search selection was shown in Figure 3. We identified 595 potentially relevant articles. After 52 duplicate records were removed, we screened the remaining 543 records for eligibility and excluded 495 publications based on titles and/or abstracts, mainly because they were reviews, conference abstracts, case reports, editorials, comments, letters, and animal experiments. 48 full-text publications were obtained for further review. 35 articles were excluded which were irrelevant to the principle of PICOS (Patients-Intervention-Comparison-Outcome-Study Style): the characteristics of patients (1 study), intervention (6 studies), comparison (10 studies), outcome (2 studies), study style (12 studies), and duplicate reports (4 studies). Therefore, only 13 RCTs were included in our meta-analysis [24–36]. Characteristics of included studies were shown in Table 1.

All these included studies were carried out in the Chinese population and published in a Chinese journal. A total of 1179 cases were enrolled in this meta-analysis. Sample size varied from 61 to 240. The participants were randomly assigned to fire-needle group and control group with their mean age ranging from 46 to 73. The duration of the treatment ranged from 2 weeks to 2 months. All the studies used fire-needle moxibustion as the intervention of treatment group. The details of interventions of control group were summarized as follows: 10 RCTs used filiform-needle acupuncture [32–36] and 3 RCTs used warm-needle moxibustion as the control [29–31]. All the included studies reported both recovery rate and recovery and marked-improvement rate to evaluate curative effect. Six studies had reported pain intensity: two studies used ISOA [28, 34], two studies adopted VAS [29, 36], and two studies employed WOMAC [30, 32]. The adverse events were only reported in two studies [28, 33], while those 11 studies did not demonstrate this [24–27, 29–32, 34–36].

### 3.2. Risk of Bias

**Figure 4:**
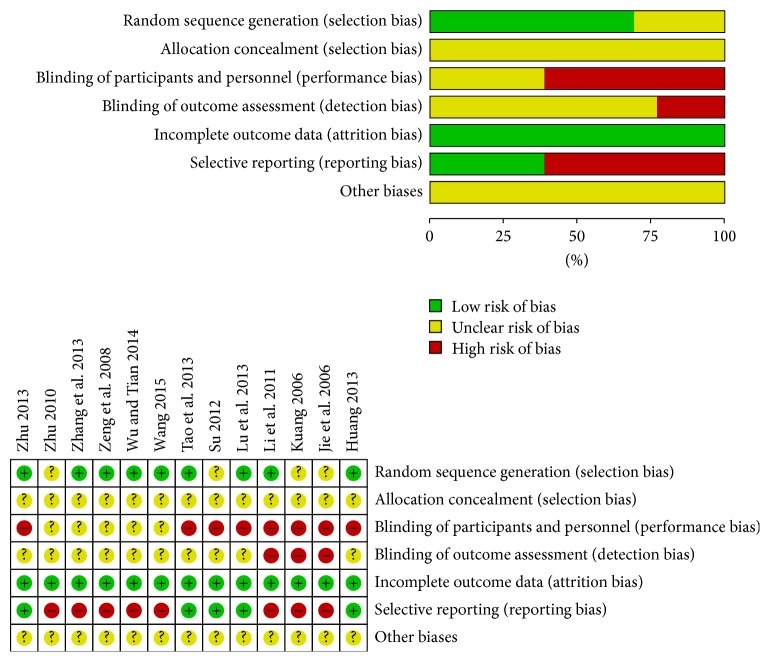
Plots of bias risk.

Risk of bias in those included studies was summarized in Figure 4. Randomization was mentioned in all trials. However, 4 studies did not report details of adequate sequence generation [24, 26, 27, 31]. The method of allocation concealment was not described in any of these trials. Three trials were considered to have a low risk of bias for blinding of outcome assessment [27, 28, 31]. Since both fire-needle patients and providers clearly were aware of the treatment, blinding of providers or patients was an impossible criterion to set for fire-needle moxibustion therapy. There was no dropout in any of the included studies, and all studies reported complete outcome data. Eight studies had a high risk of reporting bias [25–28, 30, 31, 35, 36].

### 3.3. The Recovery Rate

**Figure 5:**
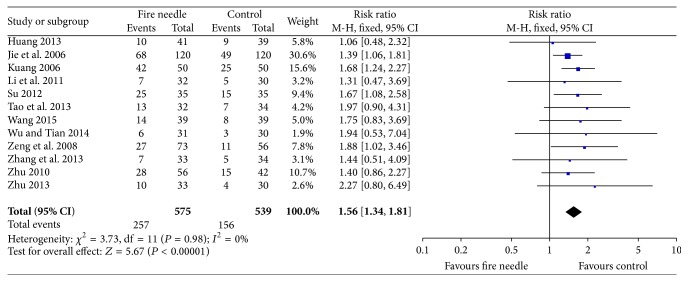
Forest plot of recovery rate.

**Figure 6:**
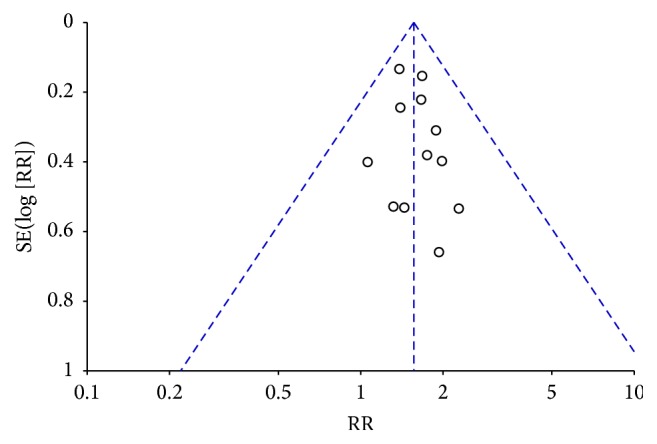
Funnel plot of recovery rate.

12 studies [24–28, 30–36] reported the recovery rate with a total sample of 1114 participants (575 in fire-needle moxibustion treatment group and 539 in the control group). A fixed-effects model was performed to analyze the data according to the heterogeneity test (*I* ^2^ = 0%, *P* = 0.98). The meta-analysis of these studies showed that there was a statistically significant difference between fire-needle group and control group in the recovery rate (RR = 1.56, 95% CI: 1.34–1.81, *P* < 0.00001; Figure 5). A funnel plot based on studies on recovery rate was generated to detect the potential publication bias, and it manifested a significant asymmetry in Figure 6 (Egger's test, *P* = 0.047).

### 3.4. The Recovery and Marked-Improvement Rate

**Figure 7:**
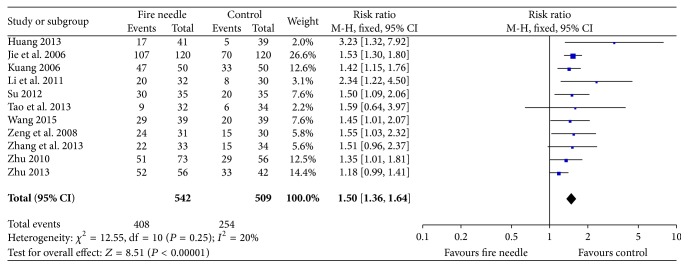
Forest plot of recovery and marked-improvement rate.

**Figure 8:**
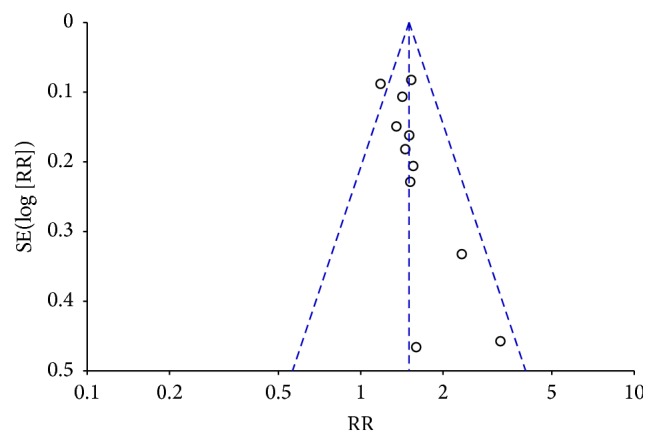
Funnel plot of recovery and marked-improvement rate.

Eleven studies involving 1051 participants (542 in fire-needle moxibustion treatment group and 509 in the control group) reported the recovery and marked-improvement rate. The data were analyzed using a fixed-effects model in accordance with the acceptable heterogeneity (*I* ^2^ = 20%, *P* = 0.25). The meta-analysis showed that there was a significant high recovery and marked-improvement rate on fire-needle moxibustion treatment compared with the control group (RR = 1.50, 95% CI: 1.36–1.64, *P* < 0.00001; Figure 7). A funnel plot analysis of 11 studies comparing fire-needle moxibustion treatment with control group on the recovery and marked-improvement rate was performed to assess the publication bias. All points in Figure 8 were asymmetrical, which indicated that publication bias might have existed (Egger's test, *P* = 0.039).

### 3.5. Pain Relief

**Figure 9:**
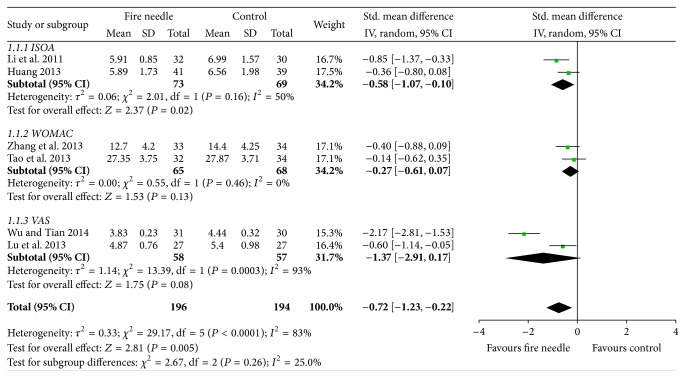
Fire-needle group and control group on pain.

Six studies measured pain intensity using VAS [29, 36], ISOA [28, 34], and WOMAC [30, 32]. A random-effects model was used because of significant heterogeneity (*I* ^2^ = 83%, *P* < 0.0001). After pooling, the results indicated that fire-needle moxibustion treatment might have a better effect on pain relief than conventional treatment (SMD = −0.72, 95% CI: −1.23–0.22, *P* = 0.005; Figure 9). We failed to conduct a funnel plot to detect publication bias on pain relief because of the insufficient number of studies.

### 3.6. Adverse Effects

Three RCTs assessed adverse effects [28, 33, 36], while those ten RCTs did not report this. Two of the three RCTs reported difficulty in movement and intolerance of cold [28, 33].

## 4. Discussion

Fire-needle moxibustion, as a kind of traditional medicine, has been widely used in China [37–39]. Although a growing number of studies reporting fire-needle moxibustion for treating KOA patients ranged from case report studies to cohort studies to randomized controlled trials, there was no systematic review specially referring to its effectiveness in the treatment of KOA. Therefore, we conducted this meta-analysis to evaluate the efficacy of fire-needle moxibustion for KOA.

Our systematic review of 13 RCTs included 1179 participants and presumably revealed that fire-needle moxibustion in the treatment of KOA might increase the recovery rate (RR = 1.56, 95% CI: 1.34–1.81, *P* < 0.00001) and recovery and marked-improvement rate (RR = 1.50, 95% CI: 1.36–1.64, *P* < 0.00001) compared with control group. In addition, the pain intensity score in the fire-needle moxibustion group was significantly lower than that in the control group (SMD = −0.72, 95% CI: −1.23–0.22, *P* = 0.005). However, GRADE profile indicated that the quality of evidence for all outcomes was relatively low. Taking into account the limited sample size and poor methodological quality of the included trials, it was difficult to draw robust conclusions.

Fire-needle moxibustion has a synergistic effect of heat from moxibustion and stimulation on acupoints in promoting calcification, improving blood circulation, and eliminating blood stasis [40, 41]. With the increasing use of fire-needle moxibustion, recent studies have reported the potential adverse events including allergies, burns, infection, difficulty in movement, and intolerance of cold [28, 33, 42]. Considering that most of the included studies have not demonstrated the adverse events and follow-up, we are unable to adequately assess the effectiveness and safety. More information is needed to better evaluate the adverse effects of fire-needle moxibustion.

The diversity in quality of the trials might lead to methodological heterogeneity, while the difference in PICO (patients, intervention, control, and outcomes) might lead to clinical heterogeneity. Subgroup analysis is an important approach to exploring the heterogeneity of treatment effects in RCTs. Given that different types of pain scale would lead to statistical heterogeneity, we conducted a subgroup analysis. However, it is difficult to assess this heterogeneity in terms of needling depth, acupuncture manipulation, and needle retention time because those detailed pieces of information are unavailable.

Our study provided the opportunity for foreign readers to recognize the advantages and exploited a new field for the research and application of fire-needle moxibustion therapy. What is more, we strictly searched the literature and thoroughly extracted and analyzed the data in order to ensure the credibility of our results.

There are some limitations that need to be considered. Firstly, the primary limitation is the poor methodological quality of the included studies. Randomization, allocation concealment, and blinding should be reported clearly, as these are the core standards of a well-designed RCT [43, 44]. The included RCTs in our study all mentioned randomization. Nevertheless, most of the trials lacked details regarding adequate sequence generation and allocation concealment. It is hard to blind the providers and patients owing to the nature of acupuncture and moxibustion. The low quality of the included studies suggested that the results should be interpreted with caution. Secondly, all trials included in the analysis were conducted in the Chinese population, which might limit the generalizability of the results. Thirdly, all trials claimed positive effects of fire-needle moxibustion in the treatment of KOA, implying that publication bias may have existed. As negative findings are less likely to be published, nonpositive studies have been inevitably missed [45]. Last but not least, we failed to generate a funnel plot for pain relief to detect potential publication bias due to the limited number of included trials.

In conclusion, the results of this meta-analysis indicate that fire-needle moxibustion may be more effective in symptom management of KOA when compared with control group. However, the findings should be interpreted cautiously because of the insufficient number of rigorously designed studies. More rigorously designed and higher quality trials with larger sample size are necessary for better elucidating the effectiveness of fire-needle moxibustion on KOA.

## Figures and Tables

**Table 1:** Characteristics of the included studies.

Author, year	Sample size (treatment/control)	Inclusion criteria ① ACR ② Guiding principles of clinical research on new drugs for TCM	Intervention of treatment group	Intervention of control group	Duration of intervention	Outcome measures (1) Curative effect ① Recovery rate ② Recovery and marked-improvement rate (2) Pain (3) Safety
Su 2012 [24]	70 (35/35)	②	Fire-needle moxibustion	Filiform-needle acupuncture	1 session = NR, twice every week, 4 times, a total of 2 sessions	(1) ①② (2) NA (3) NA

Zeng et al. 2008 [25]	129 (73/56)	①	Fire-needle moxibustion	Filiform-needle acupuncture	1 session = NR, twice every week, 4 times, a total of 2 sessions	(1) ①② (2) NA (3) NA

Zhu 2010 [26]	98 (56/42)	①	Fire-needle moxibustion	Filiform-needle acupuncture	1 session = 30 min, 3 times every week, 6 times, a total of 2 sessions	(1) ①② (2) NA (3) NA

Jie et al. 2006 [27]	240 (120/120)	②	Fire-needle moxibustion	Filiform-needle acupuncture	1 session = NR, once every 2 days, 5 times, a total of 2 sessions	(1) ①② (2) NA (3) NA

Li et al. 2011 [28]	62 (32/30)	①	Fire-needle moxibustion	Filiform-needle acupuncture	1 session = 20 min, twice every week, 16 times, a total of 2 sessions	(1) ①② (2) ISOA (3) Adverse events

Lu et al. 2013 [29]	60 (30/30)	①	Fire-needle moxibustion	Warm-needle moxibustion	1 session = NR, 3 times every week, 12 times, a total of 1 session	(1) NA (2) VAS (3) NA

Zhang et al. 2013 [30]	72 (36/36)	①	Fire-needle moxibustion	Warm-needle moxibustion	1 session = 30 min, 3 times every week, 12 times, a total of 1 session	(1) ①② (2) WOMAC (3) NA

Kuang 2006 [31]	100 (50/50)	②	Fire-needle moxibustion	Warm-needle moxibustion	1 session = NR, twice every 2 days, 10 times, a total of 3 sessions	(1) ①② (2) NA (3) NA

Tao et al. 2013 [32]	66 (32/34)	①	Fire-needle moxibustion	Filiform-needle acupuncture	1 session = 30 min, twice every 2 days, 12 times, a total of 1 session	(1) ①② (2) WOMAC (3) NA

Zhu 2013 [33]	63 (33/30)	①	Fire-needle moxibustion	Filiform-needle acupuncture	1 session = 30 min, twice every week, 12 times, a total of 1 session	(1) ①② (2) NA (3) Adverse events

Huang 2013 [34]	80 (41/39)	②	Fire-needle moxibustion	Filiform-needle acupuncture	1 session = 30 min, 3 times every week, 24 times, a total of 1 session	(1) ①② (2) ISOA (3) NA

Wang 2015 [35]	78 (39/39)	①	Fire-needle moxibustion	Filiform-needle acupuncture	1 session = 20 min, once every day, 14 times, a total of 1 session	(1) ①② (2) NA (3) NA

Wu and Tian 2014 [36]	61 (31/30)	②	Fire-needle moxibustion	Filiform-needle acupuncture	1 session = NR, 3 times every week, 10 times, a total of 3 sessions	(1) ①② (2) VAS (3) Adverse events

NA: not assessed; NR: not reported; KOA: knee osteoarthritis; ACR: American College of Rheumatology; VAS: Visual Analog Scale; WOMAC: Western Ontario and McMaster Universities Osteoarthritis Index; ISOA: Index of Severity for Osteoarthritis.

**Table 2:** Grade quality of evidence of fire-needle moxibustion treatment for KOA.

Outcome	Effect		Number of participants (studies)	Quality of the evidence (GRADE)
	Relative effect (95% CI)	Absolute effect (95% CI)		
Recovery rate	RR 1.56 (1.34 to 1.81)	162 more per 1000 (from 98 more to 234 more)	1114 (12 studies)	⊕⊕⊖ *⊖* Low^1,2^

Recovery and marked-improvement rate	RR 1.50 (1.36 to 1.64)	250 more per 1000 (from 180 more to 319 more)	1051 (11 studies)	⊕⊕*⊖* *⊖* Low^1,2^

Pain		SMD 0.72 SD lower (1.23 lower to 0.22 lower)	390 (6 studies)	⊕*⊖⊖* *⊖* Very low^1,2,3^

^1^None of the trials were blinded; most of them did not mention randomization process and allocation concealment.

^2^Published evidence is limited due to a small number of trials, all of which are showing benefits.

^3^Confidence intervals with minimal overlap; the heterogeneity is significant.
